# Corticosteroid-binding-globulin (CBG)-deficient mice show high pY216-GSK3β and phosphorylated-Tau levels in the hippocampus

**DOI:** 10.1371/journal.pone.0246930

**Published:** 2021-02-16

**Authors:** José Gulfo, Joana Pérez de San Román, Angelo Ledda, Felix Junyent, María J. Ramírez, Francisco J. Gil-Bea, Montserrat Esteve, Mar Grasa

**Affiliations:** 1 Department of Biochemistry and Molecular Biomedicine, Faculty of Biology, University of Barcelona, Barcelona, Spain; 2 CIBER Obesity and Nutrition, Institute of Health Carlos III, Madrid, Spain; 3 Institute of Biomedicine of the University of Barcelona, Barcelona, Spain; 4 Department of Pharmacology and Toxicology, University of Navarra, Pamplona, Spain; 5 Center for Applied Medical Research (CIMA), Neuroscience, University of Navarra, Pamplona, Spain; 6 Department of Cellular Biology, Physiology and Immunology, Faculty of Biology, University of Barcelona, Barcelona, Spain; 7 IdiSNA Navarra Institute for Health Research, Pamplona, Spain; Technion Israel Institute of Technology, ISRAEL

## Abstract

Corticosteroid-binding globulin (CBG) is the specific carrier of circulating glucocorticoids, but evidence suggests that it also plays an active role in modulating tissue glucocorticoid activity. CBG polymorphisms affecting its expression or affinity for glucocorticoids are associated with chronic pain, chronic fatigue, headaches, depression, hypotension, and obesity with an altered hypothalamic pituitary adrenal axis. CBG has been localized in hippocampus of humans and rodents, a brain area where glucocorticoids have an important regulatory role. However, the specific CBG function in the hippocampus is yet to be established. The aim of this study was to investigate the effect of the absence of CBG on hippocampal glucocorticoid levels and determine whether pathways regulated by glucocorticoids would be altered. We used *cbg*^*-/-*^ mice, which display low total-corticosterone and high free-corticosterone blood levels at the nadir of corticosterone secretion (morning) and at rest to evaluate the hippocampus for total- and free-corticosterone levels; 11β-hydroxysteroid dehydrogenase expression and activity; the expression of key proteins involved in glucocorticoid activity and insulin signaling; microtubule-associated protein tau phosphorylation, and neuronal and synaptic function markers. Our results revealed that at the nadir of corticosterone secretion in the resting state the *cbg*^*-/-*^ mouse hippocampus exhibited slightly elevated levels of free-corticosterone, diminished FK506 binding protein 5 expression, increased corticosterone downstream effectors and altered MAPK and PI3K pathway with increased pY216-GSK3β and phosphorylated tau. Taken together, these results indicate that CBG deficiency triggers metabolic imbalance which could lead to damage and long-term neurological pathologies.

## Introduction

Glucocorticoids, cortisol in humans and corticosterone in rodents (CORT), are endogenous steroid hormones secreted by the adrenal glands under the regulation of the hypothalamic-pituitary-adrenal (HPA) axis. They have pleiotropic functions involved in the stress response [1, 2], energy metabolism [3], reproductive function [4], and inflammatory and immune responses [5]. Excessive circulating CORT levels have been linked to insulin resistance and type 2 diabetes through their role in inhibiting the actions of insulin [6] and through impairment of pancreatic β-cell function [7, 8].

CORT also plays an important role in the central nervous system. High levels of circulating CORT are associated with memory impairment [9–11]. The effect of excess CORT on cognitive impairment is largely attributed to reduced volume of the hippocampus, deficits in neurogenesis, and CORT-mediated synaptic plasticity [12]. It has been suggested that the cognitive impairment associated with type 2 diabetes may involve CORT [13, 14], and CORT hypersecretion has been reported in Alzheimer’s disease [15, 16] with the speed of cognitive decline being linked to increases in both blood and central nervous system CORT levels at the pre-dementia clinical stage [17, 18].

The two major receptors that mediate CORT functions are the glucocorticoid receptor (GR) and mineralocorticoid receptor (MR). These receptors have different spatial distributions in the brain and peripheral organs with GR being more broadly distributed and serving as the main receptor at times of stress [19]. In the hippocampus, MR and GR coexist in the same cells [20]. GR and MR sensitivity to CORT can be modulated by FK506 binding protein 5 (FKBP5), which inhibits the receptor’s activity by delaying its translocation to the nucleus, resulting in decreased dependent transcriptional activity and thereby acting as a regulator of the HPA axis [21].

Corticosteroid-binding globulin (CBG) is a specific carrier of circulating CORT. The role of CBG is to regulate the free biologically active fraction of CORT, and approximately 80%-90% of CORT in the blood is bound to CBG with high affinity [22, 23]. However, some evidence suggests a more active role for CBG in directly modulating glucocorticoid activity [24]. In humans, several CBG mutations in the gene encoding CBG (*SERPINA6*) have been identified that affect the expression of CBG or its affinity for glucocorticoids, and some mutations knockout CBG entirely [25]. The most common clinical symptoms of patients with CBG mutations include chronic pain, chronic fatigue, chronic headaches, depression, relative hypotension, and obesity. These patients also display irregular activity of the HPA axis with low plasma levels of total-CORT and normal amounts of free-CORT, resulting in an elevated free-CORT fraction [25]. CBG-knockout mice exhibit higher mortality in response to septic shock [26], impaired response of the HPA axis to emotional stress [27], and larger adipocytes in visceral adipose tissue upon consumption of a high-fat diet [28]. Under basal conditions, CBG-deficient mice also display low total-CORT circulating levels [26, 27] with normal free-CORT levels in the evening (maximum CORT-secretion in rodents) [27] and elevated free-CORT levels at nadir CORT-secretion [26, 27]. In addition, CBG-deficient mice lose the usual sexual dimorphism of total-CORT circulating levels, and both sexes show similar values [29, 30]. Adrenal gland functionality is altered in CBG-deficient mice with diminished expression of genes involved in CORT synthesis, in spite of the increased adrenal CORT concentration in females, indicating an active role of CBG in regulating CORT adrenal excretion [31]. In contrast, it has also been shown that CBG-deficient mice present a more inflamed white adipose tissue [32]. We previously described the expression of CBG in the white adipose tissue, lung and adrenal gland [28, 30, 31, 33]. Lung CBG expression in males is greater than that in females, in contrast to the expression observed in the liver [30, 34]. In pro-inflammatory physiological environments, such as diet-induced obesity, CBG expression is increased in white adipose tissue [28]. CBG expression in the lung is also increased in the cases of acute pancreatitis and cystic fibrosis, which are characterized by serious lung inflammation [30, 34]. It has been suggested that circulating CBG may be important in delivering CORT to the brain, where it acts on neurons through fast non-genomic actions to modulate stress-induced behavior, learning, memory retrieval [35], and consolidation [36]. Furthermore, CBG has been found in different locations of the brain in humans and rodents [37–40], but its function is yet to be established.

There are two types of 11β-Hydroxysteroid dehydrogenase (11BHSD) enzymes, both of which are involved in CORT tissue bioavailability [41]. Type 1 (11BHSD1) activates CORT to convert cortisone to cortisol in humans or 11dehydrocorticosterone to corticosterone in rodents, whereas type 2 (11BHSD2) inactivates CORT, which catalyzes the reverse reaction [41]. We found that CBG-deficient mice show lower 11BHSD2 expression levels in the liver and lungs, but higher levels in visceral adipose tissue compared to those in wild-type mice [28, 30].

Based on these previous findings, the aim of the current study was to determine whether the absence of CBG would modify hippocampal CORT levels and whether pathways regulated by CORT would also be altered. For this purpose, we used *cbg*^*-/-*^ mice, which display low total-CORT and high free-CORT blood levels at the nadir of CORT-secretion under basal conditions [28]. We evaluated the hippocampus for total- and free-CORT levels, 11BHSD1 and 11BHSD2 gene expression, 11BHSD activity, and key protein expression associated with CORT activity, insulin signaling, tau phosphorylation, and neuronal and synaptic function. Our results revealed that, in the basal state, *cbg*^*-/-*^ mice exhibited slightly increased nadir free-CORT levels, elevated MR expression, increased expression of CORT target genes, reduced FKBP5 expression, altered MAPK and PI3K pathways with increased pY216-GSK3β, and greater levels of phosphorylated tau protein. There were no significant changes in the protein levels of the neuronal and synaptic function markers examined.

## Materials and methods

### Animals and experimental protocols

Sixteen-week-old wild-type (*cbg*^*+/+*^) and CBG-deficient (*cbg*^*-/-*^) male mice were used. The colony of mice was established in-house by crossing the heterozygous breeds kindly provided by Dr. T.E. Willnow [26]. The procedure used to generate CBG-knockout mice has been previously described [26]. Two mice per cage were housed in polycarbonate cages (220 mm w × 220 mm w × 145 mm h) under a controlled environment of a light cycle from 08:00 to 20:00 and in a temperature of 20–22°C. The mice were provided access to a standard laboratory pelleted formula (Teklad Global 2018, Harlan-Interfauna Ibérica, Sant Feliu de Codines, Spain) and tap water ad libitum. For the study, twelve mice of each genotype were selected and before the sacrifice were fasted overnight. The mice were weighed (36.9 ± 1.0 g *cbg*^*+/+*^ and 34.3 ± 1.2 g *cbg*^*-/-*^) and then euthanized under isoflurane anesthesia between 07:00 and 09:00. The hippocampi were carefully removed and frozen at -80°C until use. To prevent stress, the mice were kept in a separate room different from which they were anesthetized and euthanized individually. To avoid HPA activation, the handling from the cage to the sample obtention lasted no more than two minutes, and the time between the sacrifice of both mice from the same cage did not exceed 5 minutes. All procedures were conducted in accordance with the guidelines for the use of experimental animals established by the European Union, Spain, and Catalonia, and were approved by the Animal Handling Ethics Committee of the University of Barcelona.

### Determination of total and free corticosterone in the hippocampus

Hippocampal fractions of approximately 10 mg were used for lipid extraction and subsequent determination of CORT levels using a Correlate-EIA Corticosterone Enzyme Immunoassay Kit (Assay Designs, Inc., Ann Arbor, MI, USA). The hippocampal fractions were homogenized in Assay Buffer Concentrate 15® assay buffer provided in the kit. Homogenates were then sonicated on ice for 5 s at 200 W (Branson Sonifier 250® Analog Ultrasonic, Branson Ultrasonics, MI, USA) in ice. The homogenates were centrifugated at 10,000 g at 4°C for 20 min and the supernatants collected and frozen at -80°C until further use. A portion of the supernatant was used to determine the levels of free-CORT levels while the other portion was used to determine the total-CORT levels after displacement treatment. For total-CORT determination, the supernatant samples used were incubated with Steroid Displacement Reagent® (1:40) provided with the kit for 15 min to release the steroids bound to the proteins. For lipid extraction ethyl acetate (1:1) was then added to both, the aliquot for total-CORT and for free-CORT, the mixture was stirred, and the upper phase corresponding to the fat-soluble phase was extracted and collected. This extraction process was repeated three times. Finally, the samples were desiccated at -20°C on carbonic dry ice overnight and resuspended in assay buffer. The samples were stored at -80°C until use. For quantitative measurement of CORT in the hippocampus, the commercially available competitive enzyme immunoassay Correlate-EIA Kit (Assay Designs) was used. The kit contained a polyclonal antibody with high specificity to free-CORT present in standards (20,000, 4,000, 800, 160, and 32 pg/mL CORT) or in biological samples. The sensitivity of the assay was 26.99 pg/mL. The Correlate-EIA assays were performed according to the supplier’s specifications. Briefly, 200 μL of each sample and standard were included. For the B_0_ standard (0 pg/ml) and nonspecific binding (NSB) control, 100μL and 150 μL of the standard diluent (Assay Buffer 15®) were added, respectively. The corticosterone solution conjugated to alkaline phosphatase (Corticosterone EIA Conjugate®) and anti-CORT antibody (Corticosterone EIA Antibody®) were added to all wells except the NSB control well. After incubation for 2 h at room temperature with shaking (400 rpm), the wells were washed three times using Wash Buffer Concentrate®. Then, 200 μL of p-Npp Substrate® solution was added to all wells and the plate was incubate for an additional 1 h at room temperature without shaking. Immediately after the final incubation, Stop Solution® (50 μL) was added to all wells and the absorbance was read at 405 nm using a Multiskan Thermo LabSystem spectrophotometer (Thermo Scientific, MA, USA). All samples and standards were measured in duplicate.

### 11β-hydroxysteroid dehydrogenase activity in hippocampal homogenates

To evaluate 11BHSD activity, an assay mixture was used containing 100 nM ^3^H-corticosterone (specific activity 16.6 GBq/mmol; Perkin Elmer, MA, USA) in Krebs Ringer buffer (pH 7.4), 2 mM nicotinamide adenine dinucleotide phosphate (NADP), 0.2% glucose, and hippocampal homogenates obtained for western blotting that were diluted with Krebs buffer to 1.5 mg protein/mL. Blanks were included by adding buffer instead of homogenates. The reaction mixes were incubated for 2 h at 37°C. Steroids were extracted using 2 mL ethyl acetate and separated by thin layer chromatography (TLC) using dichloromethane:acetone (4:1) as the mobile phase. The radioactivity in each TLC fraction was measured using standard liquid scintillation. The assay was performed in duplicate for each sample. The activity was expressed as pmol of 11-dehydrocorticosterone per mg of protein and hour of incubation.

### RNA isolation and Real Time PCR

Total RNA from 10–20 mg hippocampus samples was extracted using TRI Reagent solution (Ambion, Inc., TX, USA). The RNA was quantified using a NanoDrop ND-1000 spectrophotometer (NanoDrop Technologies, NC, USA) and its quality verified by electrophoresis. Complementary DNA (cDNA) was then synthesized using 2 μg of RNA as template, MMLV reverse transcriptase (Promega, WI, USA), and oligo-dT primers (Attendbio, Barcelona, Spain). The reaction was incubated at 72°C for 5 min followed by 42°C for 60 min and then stored at -80°C until use. Real-time PCR was conducted using SYBR Green Master Mix (Life Technologies, CA, USA) and an ABI PRISM 7900 HT system (Applied Biosystems, CA, USA) using 10 μL of amplification mixtures containing 10 ng of cDNA and 300 nM of the corresponding forward and reverse primers. Primer sequences forward and reverse used were: 11β-hydroxysteroid dehydrogenase 1 (11BHSD1) 5’-CAAGGTCAACGTGTCCATCA-3’ and 5’-TCCCAGAGATTTCCTTCATAGC-3’; 11β-hydroxysteroid dehydrogenase 2 (11BHSD2) 5’-CTCCAAGGCAGCAATAGCAC-3’ and 5’-CGTTTCTCCCAGAGGTTCAC-3’; Glucocorticoid Receptor (GR) 5′-AACCTGACTTCCTTGGGGGC-3′ and 5′-GGCAGAGTTTGGGAGGTGGT-3′; Mineralocorticoid Receptor (MR) 5′-TGGACAGAGTTGGCAGAGGTT-3′ and 5′-CCACCTTCAGAGCCTGGGAT-3′; Plasminogen activator inhibitor-1 (PAI-1) 5’-CGCCTCCTCATCCTGCCTAA-3’ and 5’-TGTGCCGCTCTCGTTTACCT-3’; Dual specificity phosphatase 1 (DUSP1) 5′-GCTGGAGGGAGAGTGTTTGT-3′ and 5′-ATACTCCGCCTCTGCTTCAC-3′; Period circadian regulator 1 (PER1) 5’-GAGGGATTTTGGCAGATGAA-3’ and 5’-GGGACAAGGGGGTTTATTGT-3’; Serum and glucocorticoid-regulated kinase 1 (SGK1) 5’-GTGTCTTGGGGCTGTCCTGT-3’ and 5’-GGTGCCTTGCCGAGTTTGT-3’; FK506 Binding Protein 5 (FKBP5) 5′-GGCGAGGGATACTCAAACCCA-3′ and 5′-CAACGAACACCACATCTCGGC-3′; Insulin Receptor β (IRβ) 5′-ACCTTCGAGGATTACCTGCACA-3′ and 5′-CGCTTTCGGGATGGCCTACT-3′; Microtubule-associated protein tau (Tau) 5′-GCAACGTCCAGTCCAAGTGTG-3′ and 5′-CTCAGGTCCACCGGCTTGTA-3′ and for β-actin 5′-ACTGCTCTGGCTCCTAGCAC-3′ and 5′-GAGCCACCGATCCACACAGA-3′. Reactions were performed in duplicate and threshold cycle values were normalized to β-actin gene expression. Specificity of the products was determined by melting curve analysis and the ratio of the relative expression of target genes to β-actin was calculated using the ΔC(t) formula.

### Western blot analysis of hippocampal homogenates

Hippocampal samples for western blot analysis were prepared by homogenizing hippocampal tissue (10 mg) at 4°C in HEPES-buffered saline (100 mM HEPES, 200 mM NaCl, 2 mM Na4P2O7, 10% glycerol, and 5 mM EDTA, pH 7.2) containing 1% Nonidet P-40 (Roche, Basel, Switzerland) and Complete Protease Inhibitor Cocktail (diluted 1:100; Roche). Samples were sonicated on ice for 5 s at 200 W using the Branson Sonifier 250® Analog Ultrasonic sonicator. After centrifugation at 10,000 × g and 4°C for 20 min, the pellet was discarded and supernatant collected and stored at -80°C until use. Protein concentration was measured using the bicinchoninic acid (BCA) Protein Assay (Thermo Scientific, MA, USA). Samples (10–30 μg of protein) were separated electrophoretically by SDS-PAGE and electrotransferred to a polyvinylidene fluoride membrane (Millipore Corporation, MA, USA). For each group, hippocampal homogenate samples from 12 mice were transferred to two membranes, alternating two samples from *cbg*^*+/+*^ mice with 2 samples from *cbg*^*-/-*^ mice, except for CBG and DUSP1 where six hippocampus samples from *cbg*^*+/+*^ mice were followed by six samples from *cbg*^*-/-*^ mice. Prestained Protein Standard (161–0318, BioRad, CA, USA) was run along with the samples on each electrophoresis gel to determine the molecular weight of the samples. The membranes were incubated in 5% nonfat milk or 2.5% bovine serum albumin (BSA) in 0.05% TBS-Tween (pH 7.4) for 60 min at room temperature to block nonspecific binding. The membranes were then incubated overnight at 4°C with one of the following primary antibodies: anti-CBG (1:1,000; LC-C39044, LifeSpan, RI, USA), anti-GR (1:1,000; sc-12763, Santa Cruz Biotechnology, CA, USA), anti-MR (1:500; Ab64457, Abcam, MA, USA), anti-PAI-1 (1:1,000; **#**PA5-79980, Thermo Fisher Scientific, MA, USA), anti-DUSP1 (1:700; sc-37384, Santa Cruz Biotechnology), anti-pY1361-IRβ (1:1,000; #3023, Cell Signaling Technology, MA, USA), anti-IRβ (1:1,000; #3025, Cell Signaling Technology), anti-Akt (1:1,000; #9272, Cell Signaling Technology), anti-p-Akt S473 (1:1,000; #9271, Cell Signaling Technology), anti-p-ERK T202/Y204 (1:1,000; #OSE00009W, Osenses, Keswick, Australia), anti-ERK (1:2,000; sc-514302, Santa Cruz Biotechnology), anti-p-JNK T183/Y185 (1:1,000; #9251, Cell Signaling Technology), anti-JNK (1:1,000; sc-7345, Santa Cruz Biotechnology), anti-p-Tau AT8 (1:1,000; #MN1020, Thermo Fisher Scientific), anti-Tau (1:100; 57780, Sigma, MO, USA), anti-pY216-GSK3β (1:1,000; ab75745, Abcam, MA, USA), anti-pS9-GSK3β (1:1,000; #9336, Cell Signaling Technology), anti-GSK3β (1:1,000; #9315, Cell Signaling Technology), anti-synaptophysin (1:50,000; MAB368, Millipore Corporation), anti-PSD95 (1:1,000; #04–1066, Millipore Corporation), anti-NR1 (1:1,000; #05–432 Millipore Corporation), anti-NR2A (1:1,000; #04–901, Millipore Corporation), anti-Arc (1:1,000; sc-15325, Santa Cruz Biotechnology), or anti-mBDNF (1:500; VPA00760, AbD Serotec). Anti-β-actin (1:2,000; sc-47778, Santa Cruz Biotechnology or 1:10.000 A-5316 Sigma-Aldrich, MO, USA.) was used as a loading control. The immunoreactive proteins were then detected by anti-rabbit or anti-goat horseradish peroxidase-conjugated secondary antibody (1:2000; sc-2054 or 1:20,000; sc-2922, Santa Cruz Biotechnology, USA) according to the primary antibody used. Immunopositive bands were visualized with enhanced chemiluminescence (ECL) using Amersham ECL Western Blotting Detection Reagents (Amersham, Buckinghamshire, United Kingdom) or using the Luminata™ Forte Western HRP Substrate (Millipore Corporation). Optical density (OD) of the reactive bands visible on the X-ray film was densitometrically determined using ImageJ IJ1.46r free software (Wayne Rasband, National Institutes of Health). Results are expressed as the percentage of OD values relative to that of the *cbg*^*+/+*^ mice. Some of the blots were re-probed after treatment with stripping buffer, pH-6.7 (62.5mM Tris-HCl, 2% SDS, deionized water) for 15 min at 50°C.

### Statistical analysis

Data were analyzed using GraphPad software version 5.0 and are expressed as mean ± SEM. Normality was checked using Kolmogorov-Smirnov test and/or Shapiro-Wilk test depending on the sample size. Statistical comparisons were made using Student’s t-test or Mann-Whitney test with two-tailed, and P < 0.05 was considered statistically significant.

## Results

**Fig 1:**
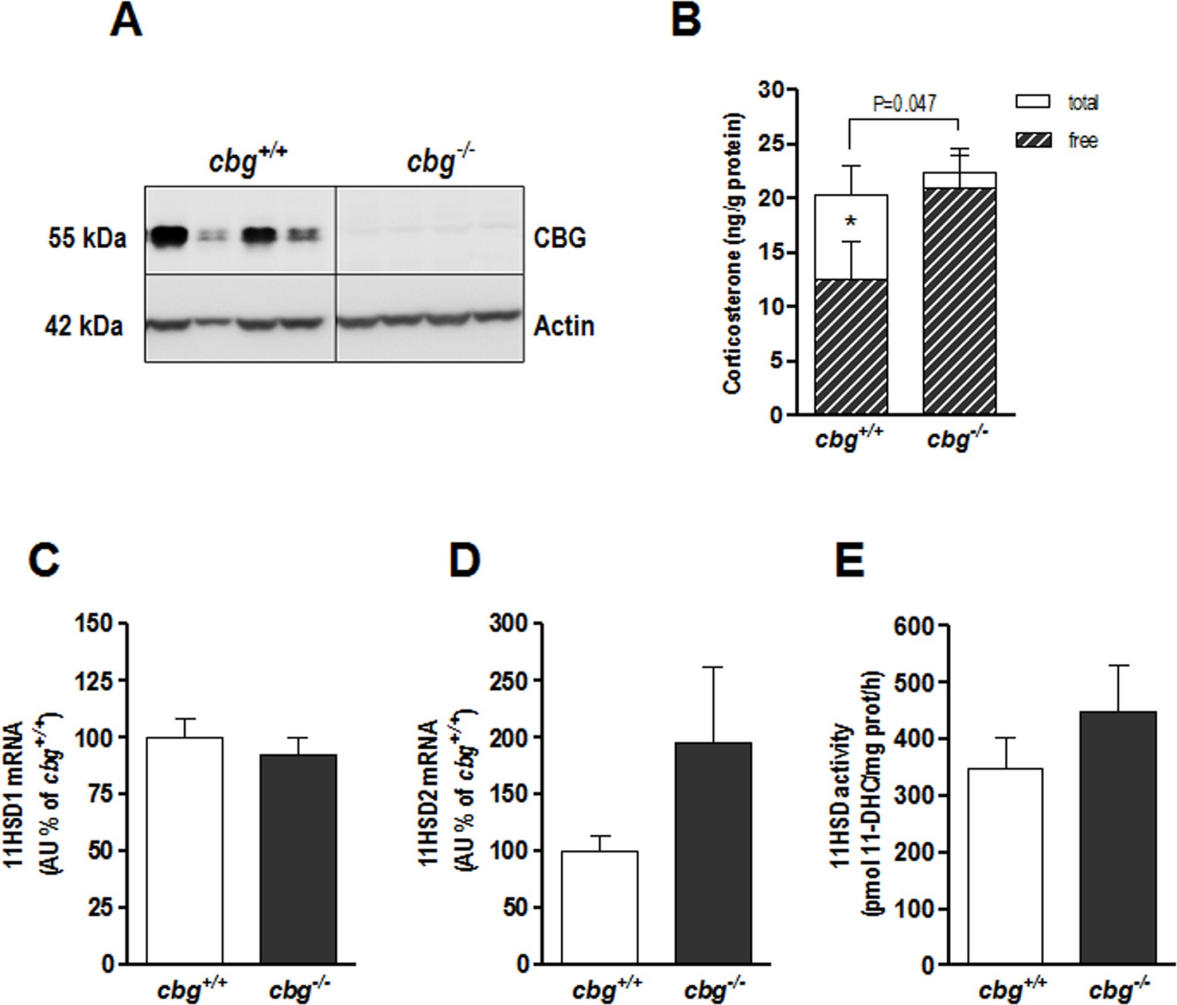
From hippocampal samples of *cbg*^*+/+*^ and *cbg*^*-/-*^ mice: A) Representative western blot of CBG, B) Total and free-CORT, C) mRNA levels of 11BHSD1, D) mRNA levels of 11BHSD2 and E) 11BHSD activity. 11-DHC = 11-Dehydrocorticosterone. Western blots show 4 representative samples for each genotype. Data are the mean ± SEM of 6–12 mice and differences between *cbg*^*+/+*^ vs *cbg*^*-/-*^: *P<0.05.

Fig 1 shows CBG protein levels, total and free-CORT levels, 11BHSD1 and 11BHSD2 mRNA levels and 11BHSD activity in hippocampal homogenates of *cbg*^*-/-*^ and *cbg*^*+/+*^ mice. The results confirmed the presence of CBG in the hippocampi of *cbg*^*+/+*^ mice, while it was absent in the hippocampal homogenates of *cbg*^*-/-*^ mice (Fig 1A). Total-CORT levels in the hippocampus did not change as a consequence of CBG deficiency (20.34 ± 2.62 vs. 22.31 ± 2.28 ng CORT/g protein in *cbg*^*+/+*^ and *cbg*^*-/-*^ mice respectively, P = 0.512), but the free-CORT levels were slightly elevated in *cbg*^*-/-*^ mice compared to those in *cbg*^*+/+*^ mice (20.90 ± 3.11 vs. 12.61 ± 3.45 ng CORT/g protein respectively, P = 0.047) (Fig 1B). There were no differences in 11BHSD activity (449 ± 82 vs. 349 ± 56 pmol 11-Dehydrocorticosterone /mg protein, P = 0,699) (Fig 1E) and neither 11BHSD1 (93 ± 7 vs. 100 ± 9, P = 0.273) and 11BHSD2 mRNA levels (195 ± 67 vs. 100 ± 15, P = 0.222) between *cbg*^*-/-*^ and *cbg*^*+/+*^ mice (Fig 1C and 1D).

**Fig 2:**
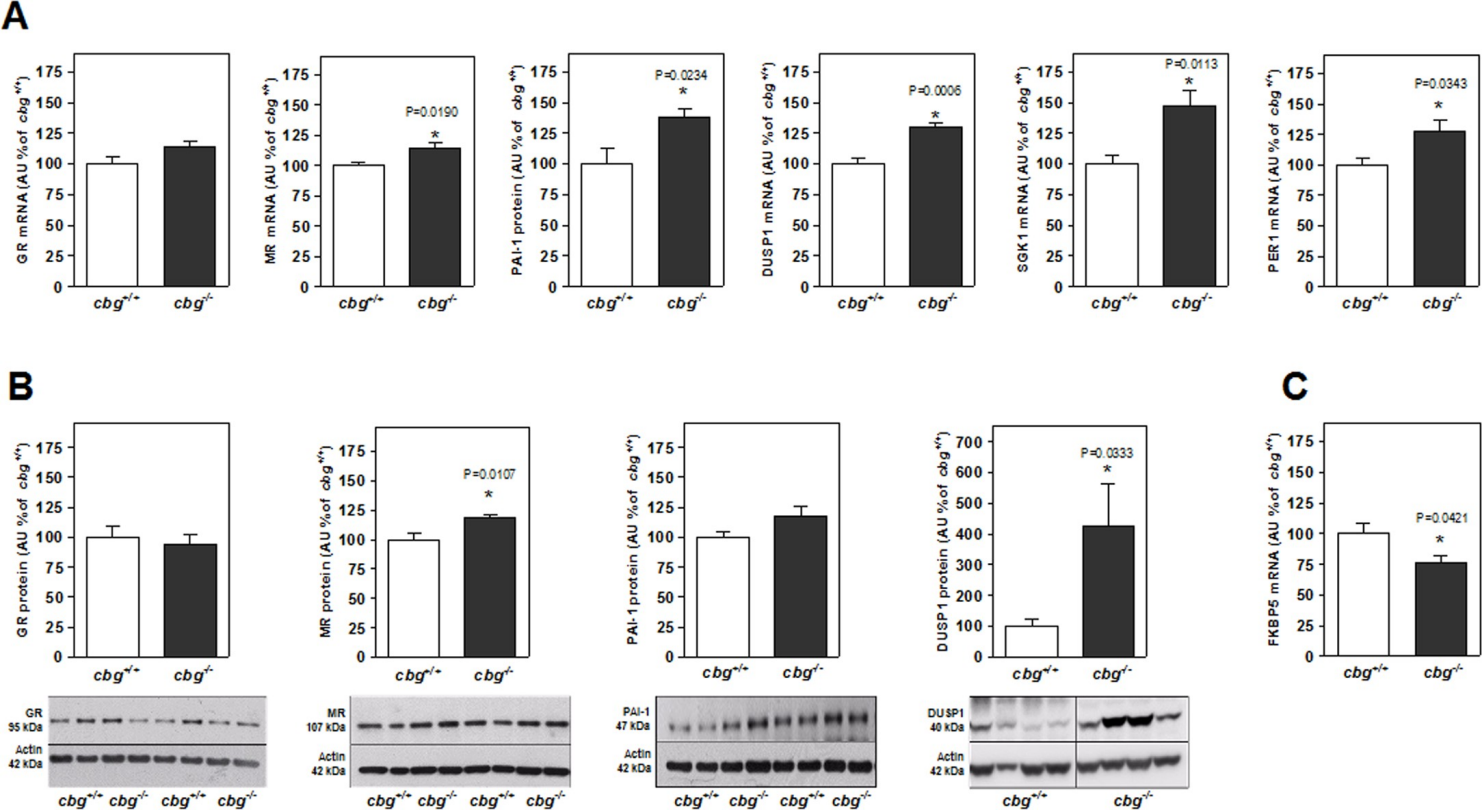
From hippocampal samples of *cbg*^*+/+*^ and *cbg*^*-/-*^ mice: A) mRNA levels of GR, MR and their target genes PAI-1, DUSP1, SGK1 and PER1, B) protein levels by western blot of GR and MR and their target gene PAI-1 and DUSP1, C) mRNA levels of the regulator of CORT activity FKBP5. All western blots show 4 representative samples for each genotype. GR = Glucocorticoid Receptor MR = Mineralocorticoid Receptor, PAI-1 = Plasminogen activator inhibitor-1, DUSP1 = Dual specificity phosphatase 1, SGK1 = Serum and glucocorticoid-regulated kinase 1 and PER1 = Period circadian regulator 1 and FKBP5 = FK506 Binding Protein 5. Data are the mean ± SEM of 6–12 mice and differences between *cbg*^*+/+*^ vs *cbg*^*-/-*^:*P<0.05.

Fig 2A shows mRNA levels of the two types of CORT receptors and their target genes PAI-1, DUSP1, SGK, and PER-1. The GR, MR, PAI-1, and DUSP1 respective protein levels were determined by western blotting (Fig 2B). The mRNA levels of FKBP5, a regulator of CORT activity, are shown in Fig 2C. The levels of GR mRNA and protein were unchanged in the hippocampus of *cbg*^*-/-*^ mice compared to those of *cbg*^*+/+*^ mice, but MR mRNA and protein levels were significantly increased in CBG-deficient mice. FKBP5 expression was reduced in *cbg*^*-/-*^ mice, which allowed CORT action through GR and MR with increased DUSP1, PAI-1, SGK1 and PER-1 expression, all of which are downstream genes regulated by CORT through GR, MR, and GR/MR activity.

**Fig 3:**
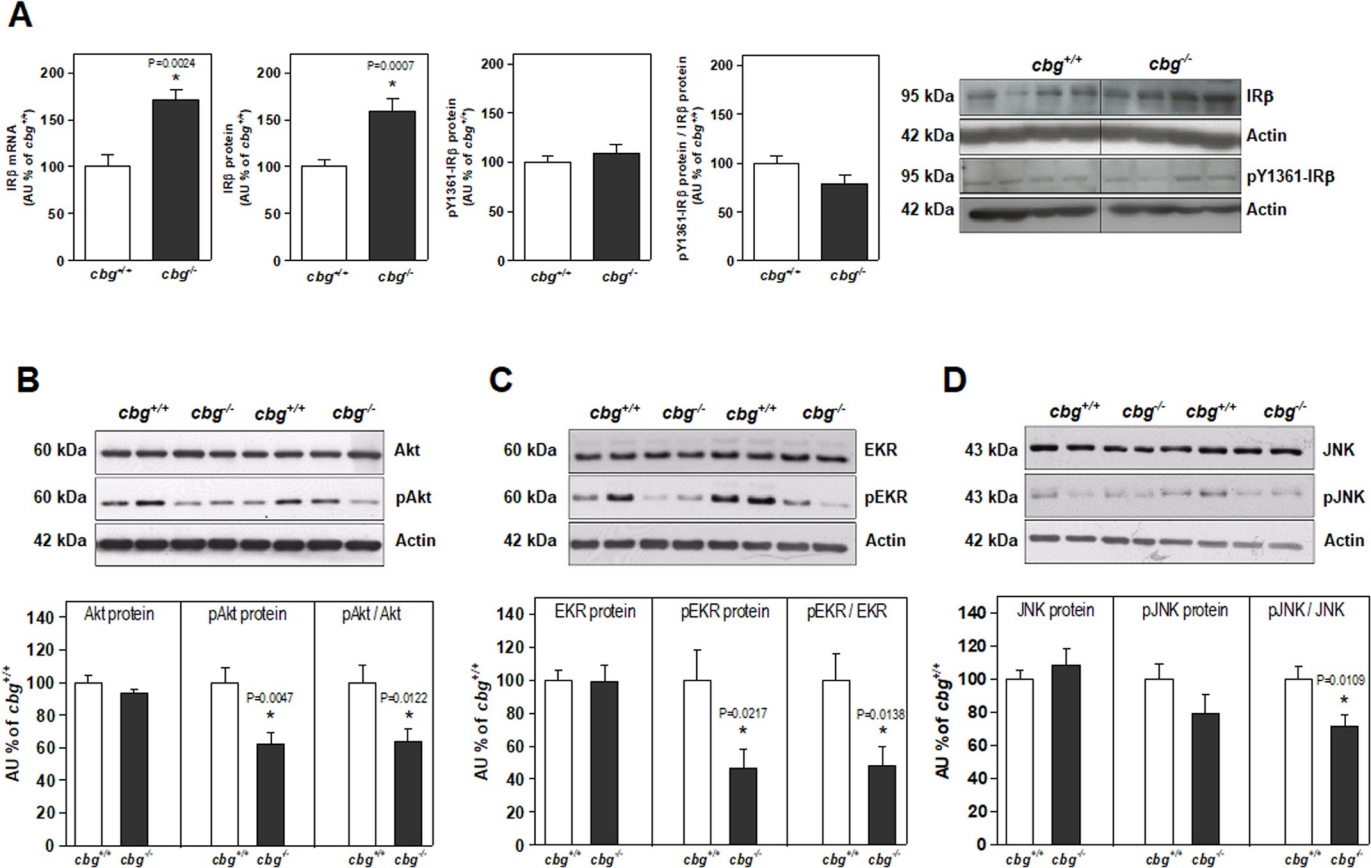
From hippocampal samples of *cbg*^*+/+*^ and *cbg*^*-/-*^ mice: A) IRβ mRNA levels and total IRβ and phosphorylated IRβ (tyr-1361) protein levels; B) Total Akt and phosphorylated Akt protein levels; C) ERK and phosphorylated ERK protein levels; D) JNK and phosphorylated JNK protein levels. All western blots show 4 representative samples for each genotype. Data are the mean ± SEM of 6–12 mice and differences between *cbg*^*+/+*^ vs *cbg*^*-/-*^: *P<0.05.

IRβ expression and its phosphorylation at residue tyrosine-1361, which are associated with IR transduction through IRS1/2 to PI3K or MAPK signaling, are shown in Fig 3A. Increased levels of IRβ mRNA and protein were found in *cbg*^*-/-*^ mice compared to those in *cbg*^*+/+*^ mice; however, there were no changes in total phosphorylation levels (pY1361IRβ). Consequently, was observed a tendency of decreased pY1361IRβ/IRβ ratio, but this was not statistically significant. Downstream effectors of the IR-PIK3 pathway, such as Akt, showed no changes in the levels of total protein in the hippocampus of *cbg*^*-/-*^ mice, but did demonstrate reduced phosphorylation (Fig 3B). A similar pattern was observed for downstream effectors of the IR-MAPK pathway, including ERK and JNK (Fig 3C and 3D). Taken together, these data indicate that the insulin signaling pathway in *cbg*^*-/-*^ mice was altered compared to that in *cbg*^*+/+*^ mice.

**Fig 4:**
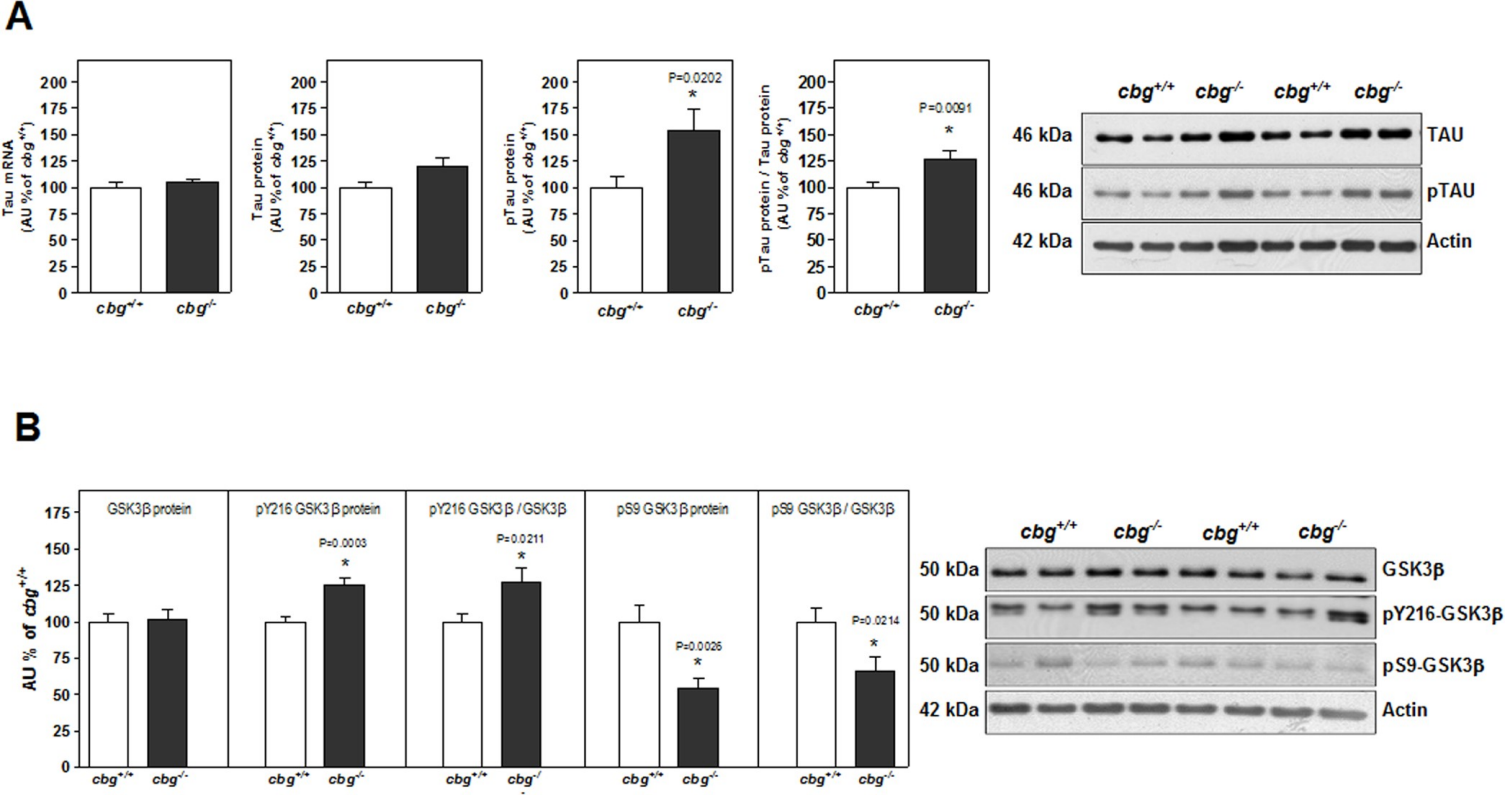
From hippocampal samples of *cbg*^*+/+*^ and *cbg*^*-/-*^ mice: A) Tau mRNA levels, total Tau and phosphorylated Tau protein levels; B) Protein levels of GSK3β, phosphorylated GSK3β (tyr-216) active form and phosphorylated GSK3β (ser-9) inactive form. All western blots show 4 representative samples for each genotype. Data are the mean ± SEM of 6–12 mice and differences between *cbg*^*+/+*^ vs *cbg*^*-/-*^: *P<0.05.

No differences in Tau mRNA or total protein levels were found between *cbg*^*+/+*^ and *cbg*^*-/-*^ mice; however, CBG deficiency was associated with a significant increase in the phosphorylation of Tau protein at residues serine-202 and threonine-205, both of which were detected by the anti-AT8 antibody (Fig 4A). The active form of GSK3β, which is phosphorylated at residue tyrosine-216, was elevated in *cbg*^*-/-*^ mice, while levels of the inactive form, which is phosphorylated at residue serine-9, were reduced compared to that in *cbg*^*+/+*^ mice (Fig 4B). Lower levels of the inactive form of GSK3β were in accordance with the reduced levels of pAkt, which is responsible for GSK3β phosphorylation at serine-9 and increased levels of phosphorylated Tau (pTau).

**Fig 5:**
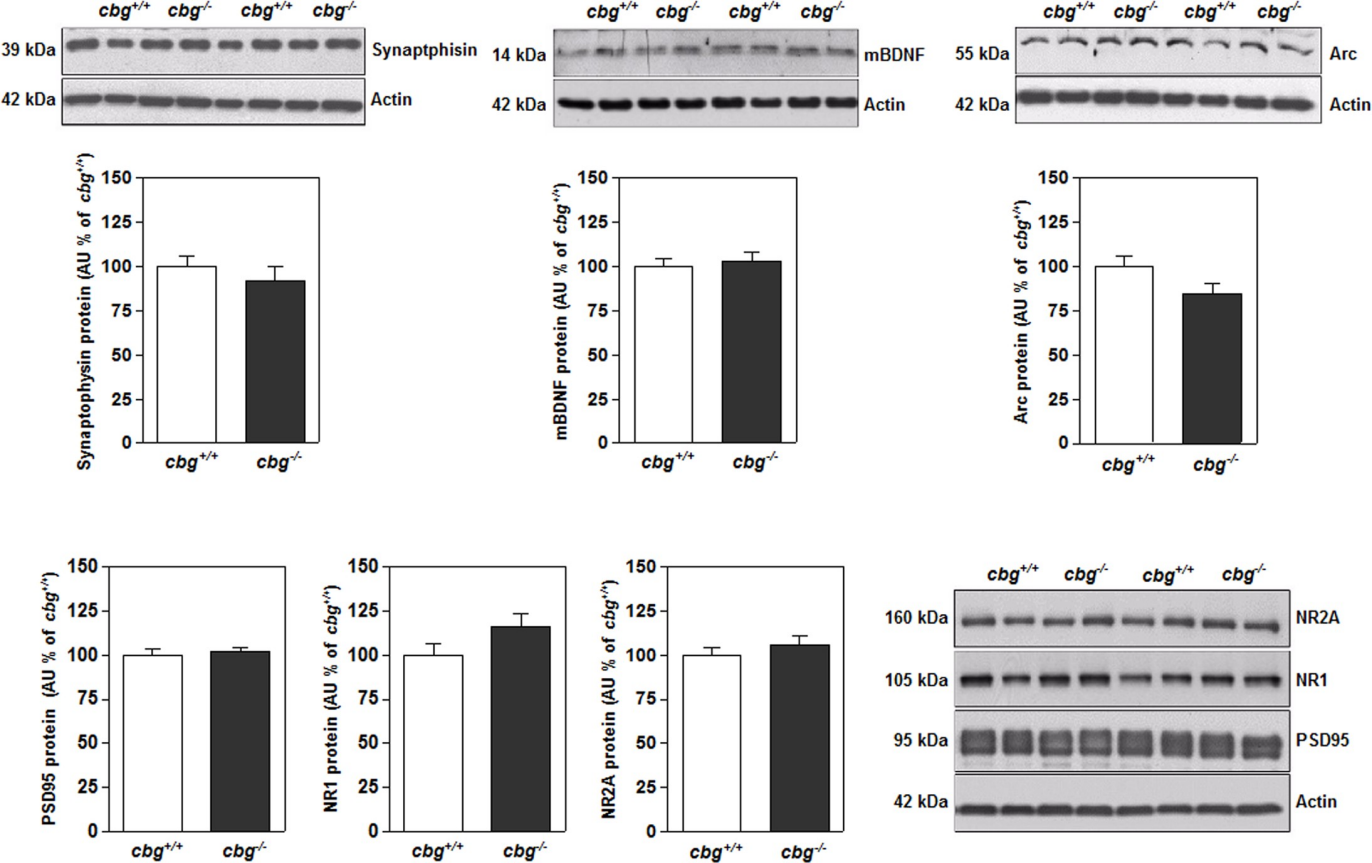
From hippocampal samples of *cbg*^*+/+*^ and *cbg*^*-/-*^ mice: synaptophysin, mBDNF, arc, PSD95, NR1 and NR2A protein levels. All western blots show 4 representative samples for each genotype. Data are the mean ± SEM of 6–12 mice and differences between *cbg*^*+/+*^ vs *cbg*^*-/-*^: *P<0.05.

Fig 5 shows the protein levels of neuronal and synaptic function markers. Although CBG deficiency altered insulin signaling and Tau phosphorylation in the hippocampus of *cbg*^*-/-*^ mice, no significant differences in the protein levels of the neuronal and synaptic function markers evaluated were detected between *cbg*^*+/+*^ and *cbg*^*-/-*^ mice.

## Discussion/Conclusion

The current study investigated whether CBG deficiency affected glucocorticoid levels and activity in the hippocampus, an area of the brain that is involved in learning and memory processes and contains a high density of GR and MR [20, 42] and, in which CORT plays an important role [10–12]. The presence of CBG has been reported in cells from the pineal gland, hypothalamus, hippocampus, and cerebrospinal fluid [37–40], although its role is currently unknown. Here, we confirmed the presence of CBG protein in the mouse hippocampus and found that at rest, CBG deficiency resulted in slightly elevated morning levels of free-CORT and features typical of CORT excess, suggesting that CBG regulates CORT availability in this part of the nervous system. Tissue bioavailability of CORT is regulated through 11BHSD activity. In the present study, we measured 11BHSD1 and 11BHSD2 expression and the overall 11BHSD activity. It is difficult to specifically determine type 1 activity because ^3^H-11-dehydrocorticosterone is not commercially available. It has been previously reported that the hippocampus is one of the brain areas that is rich in 11BHSD1 activity [43], where it acts as a reductase [44] providing corticosterone, and is responsible in part for circadian and stress corticosterone fluctuations in the hippocampus [45]. Although we previously reported altered local 11BHSD1 and 2 expression in tissues such as liver, lung, and white adipose tissue due to CBG deficiency [28, 30], herein we identified no differences in the hippocampus. According to our results, while plasma total-CORT levels are lower in *cbg*^*-/-*^ mice, the similar hippocampal total-CORT levels compared to *cbg*^*+/+*^ mice cannot be explained by locally increased 11BHSD activity.

The knockout mouse model used in the present study exhibits reduced serum levels of total-CORT compared to those in *cbg*^*+/+*^ mice (90-175nM in *cbg*^*-/-*^ vs. 200-300nM in *cbg*^*+/+*^) and higher amounts of free-CORT compared to *cbg*^*+/+*^ mice (10-30nM in *cbg*^*-/-*^ vs. 3-5nM in *cbg*^*+/+*^) measured at the nadir of the circadian rhythm and under basal conditions as previously reported [26, 28, 30, 31]. In contrast, we observed similar levels of total-CORT, but significantly higher free-CORT levels in the hippocampus of *cbg*^*-/-*^ mice compared to those in *cbg*^*+/+*^ mice, which represented 93% of the total CORT. However, other authors have reported reduced levels of free-CORT in the dorsal hippocampus of CBG-deficient mice after exposure to stress [35]. Differing experimental conditions can explain these discrepancies. In the present study, hippocampal CORT content was evaluated from a tissue homogenate at a specific point, in the morning (the nadir point of CORT secretion), and under basal conditions, whereas Minni et al. [35] evaluated the CORT level over time through microdialysis of a cannulated dorsal hippocampus before and after a stress test. Similar corticosterone levels have also been found in the adrenal gland of *cbg*^*-/-*^ compared to *cbg*^*+/+*^ mice, particularly in females. This was found despite the downregulation in the expression of enzymes involved in CORT synthesis as a consequence of CBG deficiency, suggesting a role of CBG in mediating tissue CORT release [31]. We investigated whether *cbg*^*-/-*^ mice hippocampi showed increased glucocorticoid activity. While there were no differences in the levels of GR, increased MR expression was observed. In the same way, Solas et al. found that in situations of glucocorticoid excess, there was a decrease in GR but an increase in MR levels [46, 47] which is consistent with our results. This implies that different levels of CORT may elicit different responses by binding to the MR and/or GR, leading to the formation of homodimers (GR-GR) or heterodimers (GR-MR) that trigger the expression of different responsive genes, and thereby, different signaling pathways [46, 48, 49]. Datson et al. reported that CORT in the hippocampus mediates its actions mainly through either the MR or GR, while only a few targets are responsive to both MR and GR activation [50]. Here, consistent with elevated CORT activity being regulated through the MR, we observed increased gene expression of PAI-1, a known downstream effector of activated MR [51]. However, the increased levels of DUSP1 in CBG-deficient mice, a CORT target gene induced through GR [52], suggest that GR-mediated actions are also increased in the absence of CBG. In addition, increased gene expression of SGK-1 and PER-1, GR/MR target genes [53, 54], also occurs in CBG-deficient mice. An increase in GR and MR receptor expression is normally expected when CORT levels are deficient, for example, after adrenalectomy [55]. Minni et al. [56] found a tendency of increased GR and MR mRNA levels in the hippocampus of CBG-deficient mice 3 hours after stress, which would be consistent with low CORT activity. However, Solas et al. [46, 47], as mentioned above, in mice under a CORT-excess environment found a decrease in GR but an increase in MR, approaching that found in CBG-deficient mice. On the other hand, FKBP5 is a co-chaperone that promotes receptor-complex conformation, lowering the affinity of CORT to the GR [21] and MR [57], inhibiting receptor activity, and downregulating CORT activity Thus, FKBP5 enables homeostatic regulation of the HPA axis, which is essential for the stress response [58]. In the current study, we found an unexpected decrease in FKBP5 in *cbg*^*-/-*^ mice. FKBP5 expression typically increases under the action of CORT, with a role in restraining the effects of CORT and preventing the interaction of CORT with the GR and MR [21]. Polymorphisms affecting FKBP5 affinity for CORT receptors have been associated with HPA axis disorders, such as anxiety or stress altered responses [21, 58, 59]. As noted above, CBG-deficient mice have an altered HPA axis with elevated ACTH plasma levels [26] and a diminished adrenal response that entails adrenal hyperplasia [28, 31], with impairment of CORT synthesis and secretion [31]. Collectively, our current findings, in addition to those previously reported, reinforce the hypothesis that CBG has an unexplored role in CORT action and homeostatic regulation of the HPA axis.

It has been suggested that cognitive impairment in diabetes may be linked to elevated glucocorticoid levels that are frequently associated with this disease [13]. Although a clear decrease in IRβ phosphorylation in the hippocampus of *cbg*^*-/-*^ mice was not found, a decrease in downstream IR-regulated effectors was observed, despite the significant increase in IRβ total expression. *cbg*^*-/-*^ mice exhibited alterations in the PI3K signaling pathway with reduced levels of phosphorylated Akt and thereby lower levels of pS9GSK3β, the inactive form of GSK3β. These results are in agreement with a previous study where CORT excess was shown to induce insulin resistance through increased MR expression [46]. However, we previously reported that *cbg*^*-/-*^ mice do not show altered serum glucose or insulin levels [28].

MAPK pathway activation by insulin triggers ERK and JNK phosphorylation. CBG deficiency is associated with a reduction in phosphorylated forms of both ERK and JNK. In addition, JNK is central to the stress-induced insulin resistance response, as it phosphorylates IRS1 at the inhibitory site Ser-307 and blocks insulin signal transduction [60, 61]. Previously, Solas et al. reported that chronic CORT administration elicits insulin resistance in the hippocampus by promoting JNK activation [46], the opposite to our finding in CBG-deficient mice. The main difference between these experiments is the lack of CBG. DUSP1 upregulation observed in *cbg*^*-/-*^ mice may be responsible for the decreased levels of pJNK, as DUSP1 is known to block MAPK and JNK activation [62].

Excess CORT has been linked to cognitive impairment and Alzheimer’s disease, with pTau protein also being involved [46]. In parallel, *cbg*^*-/-*^ mice had significantly increased pTau levels in the hippocampus, perhaps due to the slight increase in free-CORT levels. Tau phosphorylation is carried out by several kinases belonging to different signaling pathways, such as GSK3β. Our current results suggest that increased pTau levels in CBG-deficient mice may be a consequence of an altered Akt-Gsk3β pathway, as *cbg*^*-/-*^ mice showed increased pY216-GSK3β (active form of GSK3β) and decreased pS9-GSK3β (inactive form of GSK3β) levels, which would be expected to result in increased levels of pTau.

Despite the changes in the MAPK and PI3K signaling pathways and Tau phosphorylation, *cbg*^*-/-*^ mice did not show any apparent impairments in synaptic function, as there were no changes in the synaptic markers evaluated here. Although excess CORT promotes neurodegeneration and decreases neurogenesis through the reduction of mBDNF expression [63], we did not observe any change in the hippocampal content of mBDNF in *cbg*^*-/-*^ mice. Previous behavior experiments have shown normal short-term memory, choice latency times, and initial responses to turn alternation patterns in *cbg*^*-/-*^ mice of comparable age [35], but impaired contextual and recognition memory consolidation [36].

In conclusion, our results showed that CBG-deficiency elicits significant alterations in hippocampal CORT activity. Under basal conditions, CBG-deficient mice showed slightly elevated morning levels of free-CORT in the hippocampus, FKBP5 downregulation, high MR expression, and increased MR and GR downstream gene expression without changes in local 11BHSD expression and activity. In addition, CBG-deficient mice displayed altered MAPK and PI3K signaling pathways, with decreased pERK, pJNK and pAkt, and increased pY216-GSK3β (active form) and Tau phosphorylation. An important limitation of the present study is that CORT quantification has been made only at one specific time-point, in the nadir of CORT secretion, and at baseline. Further studies characterizing the full circadian rhythm and the response to physiological insults are required to confirm the role of CBG on central nervous system, specifically the hippocampus, and its possible involvement in the progression of neurological damage.

## Supporting information

S1 File (PDF)
