# Supplementary material for: Corticosteroid-binding-globulin (CBG)-deficient mice show high pY216-GSK3β and phosphorylated-Tau levels in the hippocampus
Source: PLoS One. 2021 Feb 16;16(2):e0246930. doi: 10.1371/journal.pone.0246930 (PMC7886218; doi:10.1371/journal.pone.0246930)

Supplemental data to reviewers

In order to facilitate the review, we include the full blots obtained from which we selected the bands shown in the figures.

The western blots performed in the Department of Pharmacology and Toxicology of the University of Navarra were organized as follows:

- For each group, samples of hippocampi homogenates from 12 mice were distributed in 2 membranes, alterning 2 samples of WT mice with 2 samples of KO mice.

The full membranes corresponding to each figure showed in the manuscript are included here.

Membranes 1 and 2 were used to: immunoblot of **MR**, **total and phosphorylated Akt**, **synaptophysin** and the corresponding **actin**.

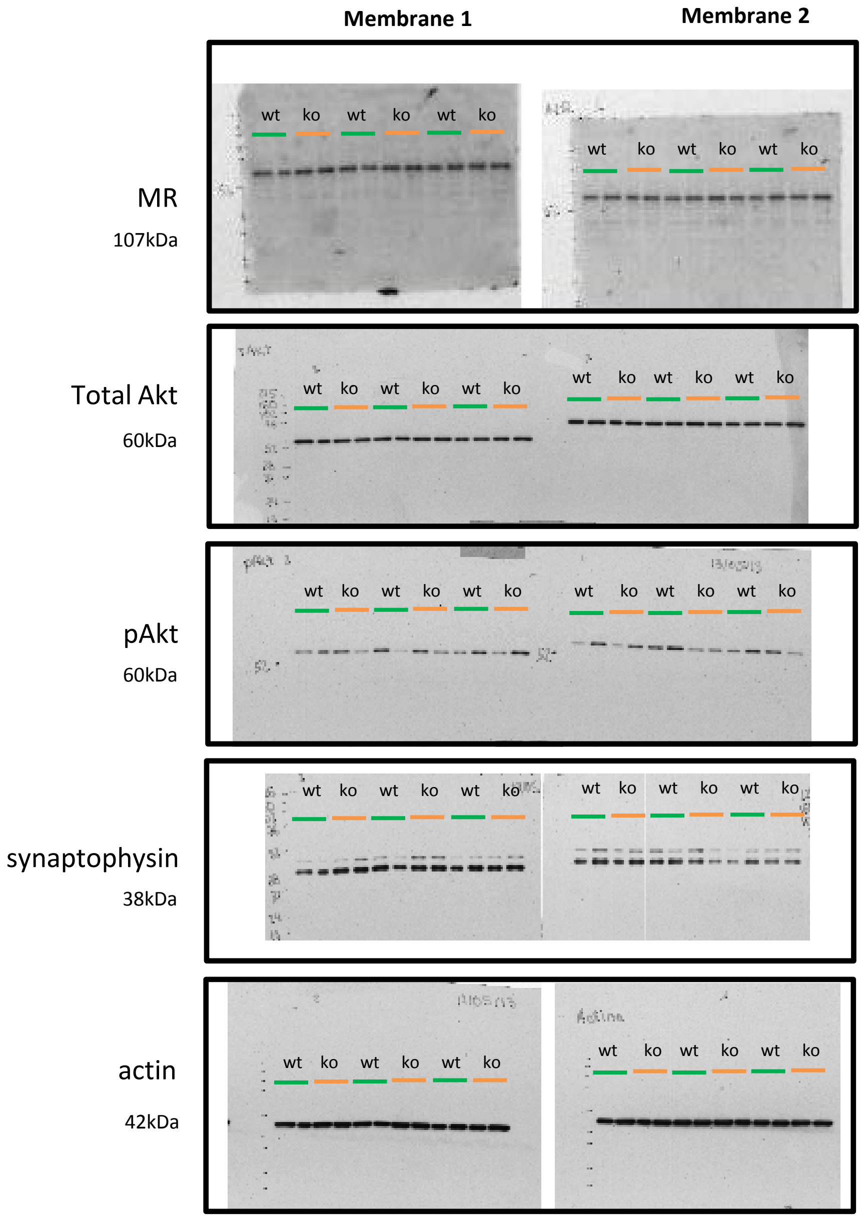

Membranes 3 and 4 were used to: immunoblot of **GR**, **PAI-1**, **total and phosphorylated ERK, total and phosphorylated JNK**, **total and phosphorylated Tau, total and phosphorylated GSK3β at pY216**, **total and phosphorylated GSK3β at pS9, mBDNF** and the corresponding **actin**.

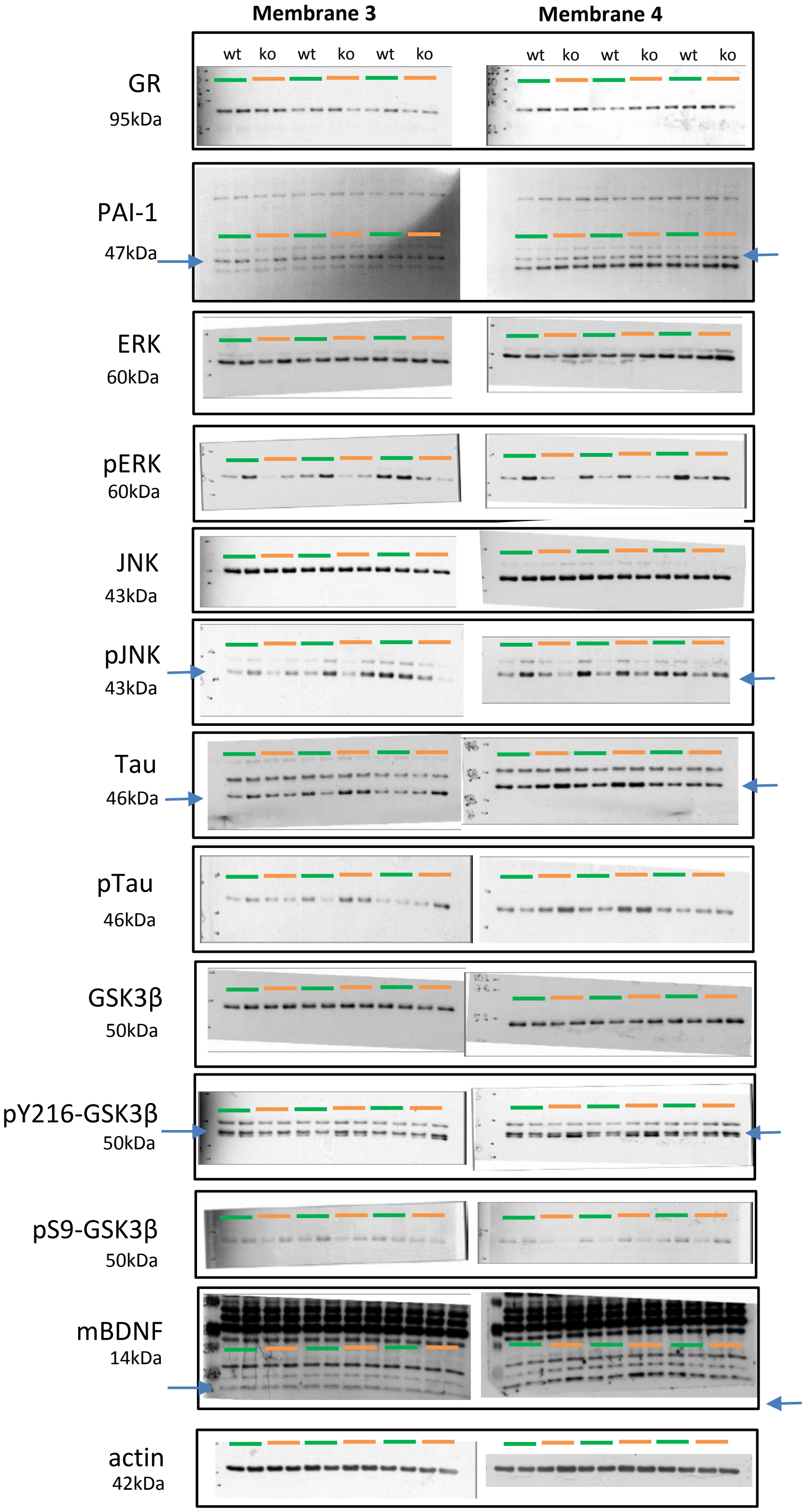

Membranes 5 and 6 were used to: immunoblot of **arc** and the corresponding **actin**.

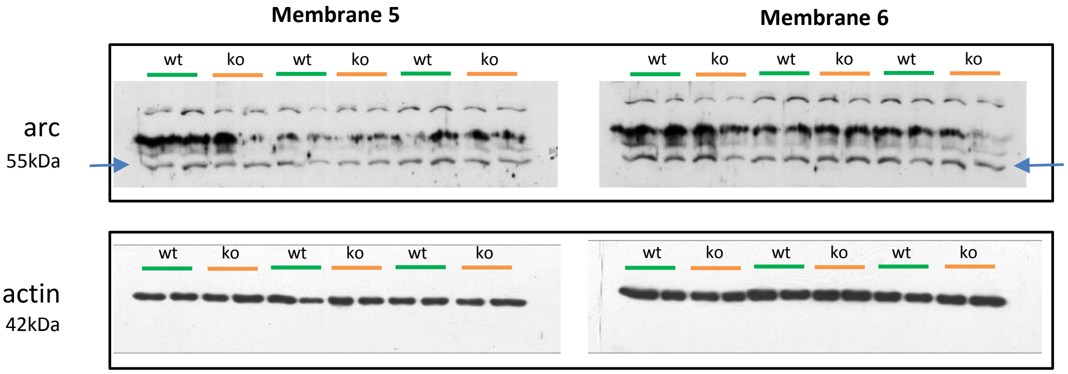

Membranes 7 and 8 were used to: immunoblot of **total and phosphorylated IRβ** and the corresponding **actin** in showed in figures of previous version of the manuscript. In the new version, we have replaced this for new blots which are shown later in this document

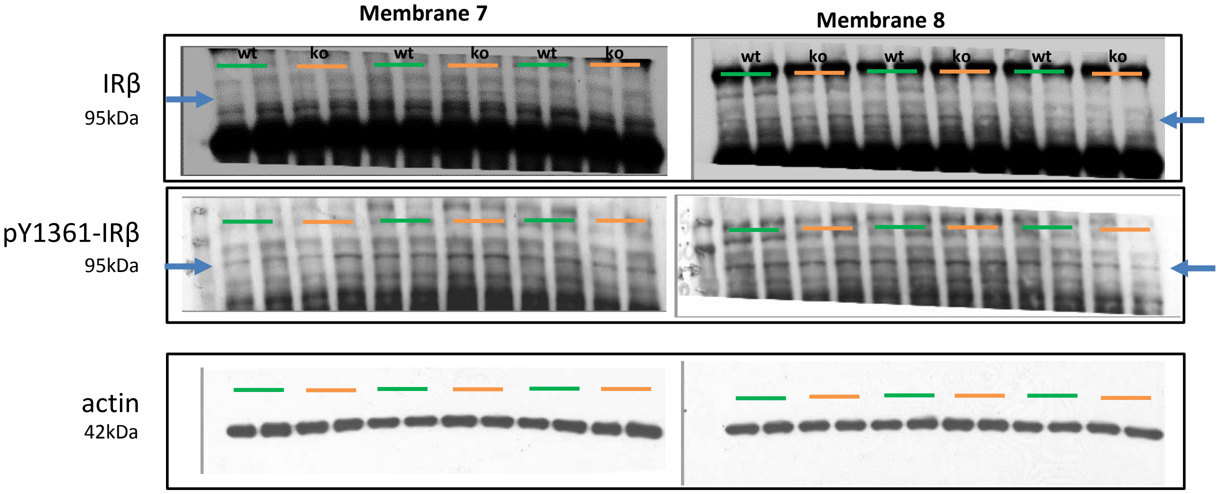

Membranes 9 and 10 were used to: immunoblot of **PSD95** (Figure 5B), **NR1** (Figure 5C), NR2A (Figure 5D) and the corresponding **actin**.

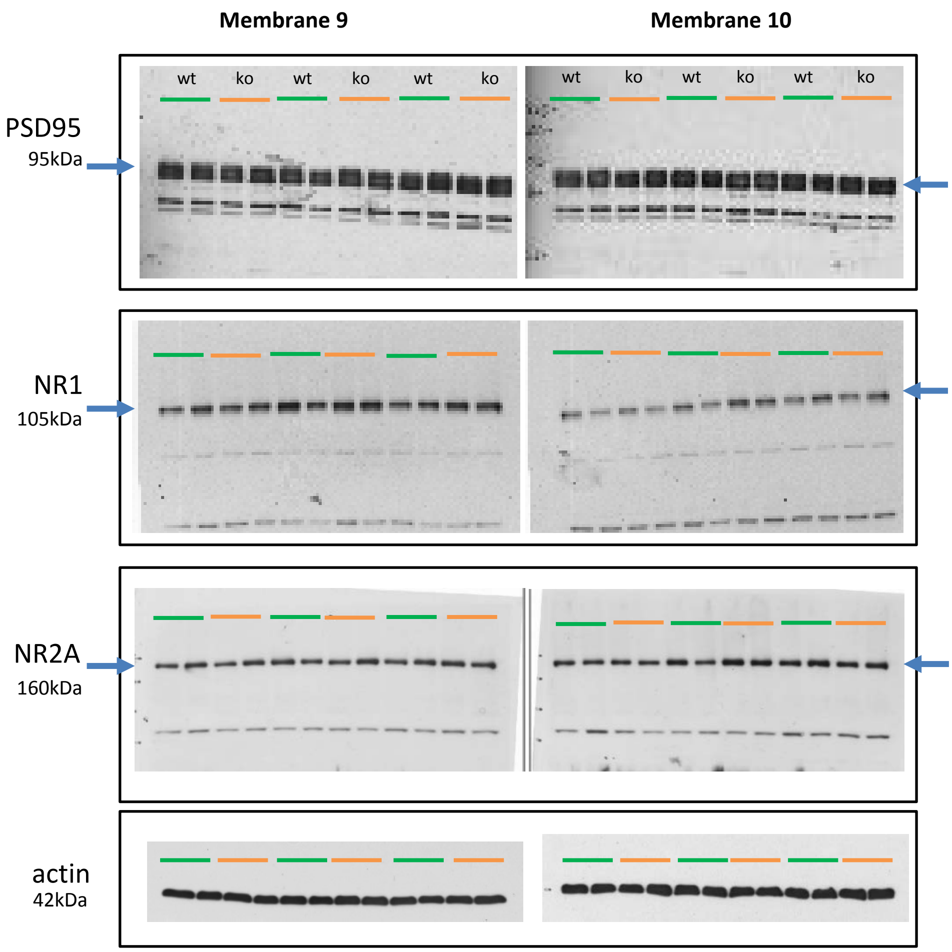

The western blots performed in the Department of Biochemistry and Molecular Biomedicine of the University of Barcelona were organized different. In this case, samples of hippocampi homogenates from 6 mice for each group were distributed in 1 membrane, alterning 6 samples of WT mice with 6 samples of KO mice.

The full membrane corresponding to each figure showed in the manuscript are included here. **Membrane 11**: immunoblot of **CBG, DUSP-1** and the corresponding **actin**.

Note that the not stripped membrane used for CBG immunoblot was then incubated with anti-DUSP1, in order to obtain the maximal signal. This is the reason CBG bands are perfectly marked over DUSP1 ones in the immunoblot of DUSP1. In addition, we want explain that, in this case was charged 5µL of homogenate in order to determinate the presences and absence of CBG (not quantify), but then we utilized it to immunoblotting DUSP-1. This fact could explain a different charge and variability of stains, but actin corrected it.

Membrane 11

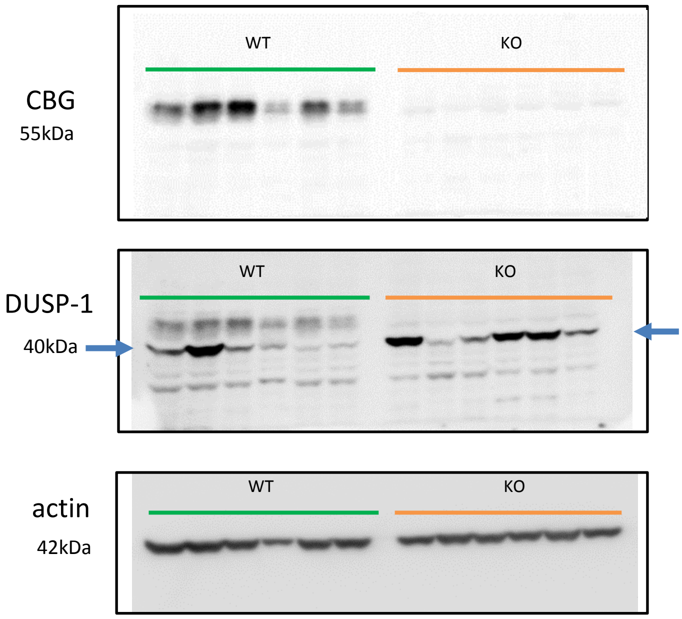

After first revision, the editor point his attention in the western blot related with Insulin receptor (IR and phosphorylated insulin receptor (pIR), that which seemed a bit unconvincing. For that reason, we repeat this western. In this case we charged 80µg of protein, is for that actin in membranes 12 and 13 has a very strong brand. These are the blots shown in the manuscript corrected.

Here we show a test of the specificity of the antibody for IR and pIR since we obtained several bands.

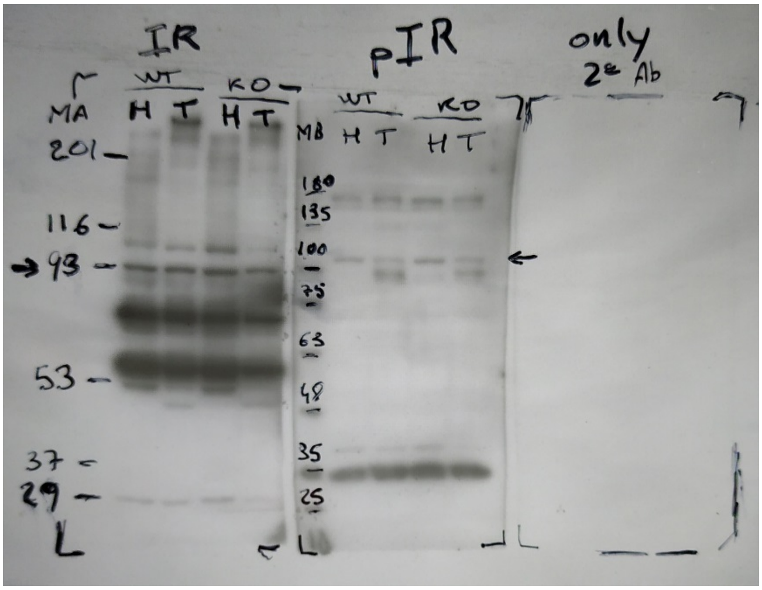

We want to point out that we no longer had all the homogenates available, so we tested with the available homogenate samples and the proteins obtained from the fraction resulting from the RNA isolation (Tri Reagent).

H= homogenate
T= protein from Tri Reagent fraction
MA= 10 µL of Prestained SDS-PAGE standard (Broad range cat 161-0318 (control 98561), BioRad)
MB= 1 µL of Prestained Protein Ladder (Broad range act ab116028, abcam)

We can see how the specific bands are obtained for IR and pIR and that when the secondary antibody only is incubated no signal was obtained.

Membrane 12: immunoblot of **IR, pIR** and the corresponding **actin**.

We observed that the samples from protein isolated with Tri Reagent had a lower signal in the case of pIR, it is possible that IR has been dephosphorylated. The band that is observed below the pIR band would support this idea. It is for this reason that we only have considered the results from homogenates that had phosphatases inhibitors.

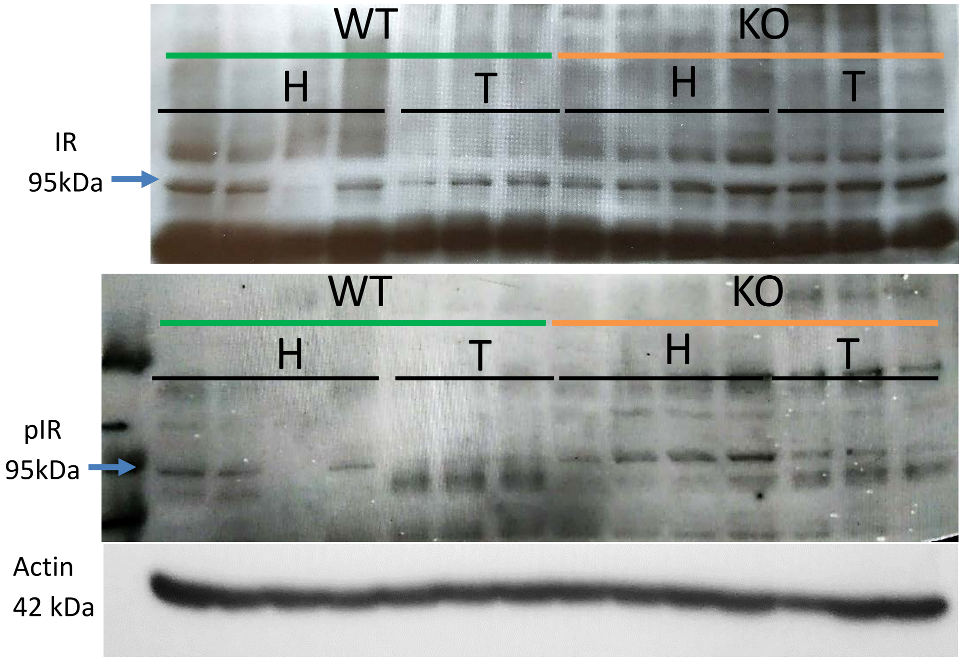

Membrane 13: immunoblot of **pIR** and the corresponding **actin**. Homogenates.

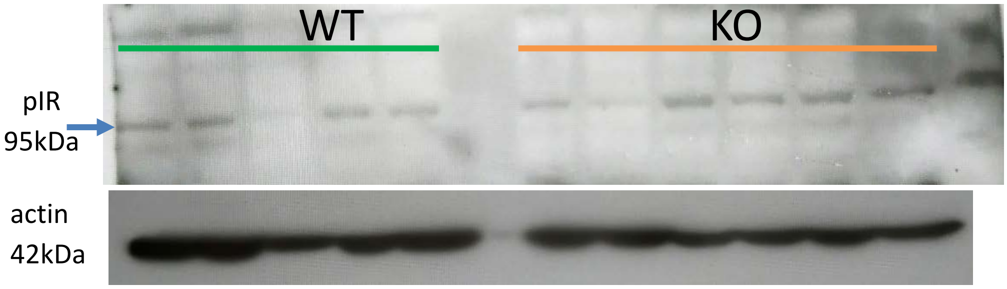

Membrane 14: immunoblot of **pIR** and the corresponding **actin**. Protein isolated with Tri Reagent

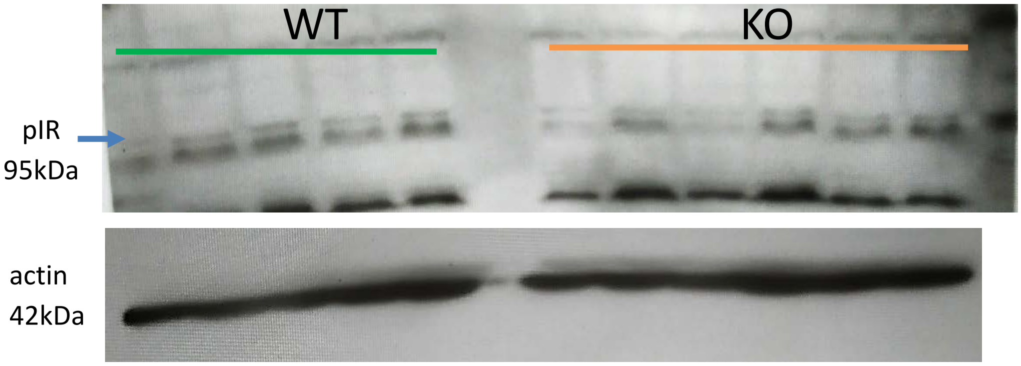

Supplement: S1 File — (PDF) [file pone.0246930.s001.pdf]
